# Evaluation on the Efficiency of Biomass Power Generation Industry in China

**DOI:** 10.1155/2014/831372

**Published:** 2014-06-26

**Authors:** Jingqi Sun, Dong Sun, Sen Guo

**Affiliations:** School of Economics and Management, North China Electric Power University, Beijing 102206, China

## Abstract

As a developing country with large population, China is facing the problems of energy resource shortage and growing environmental pollution arising from the coal-dominated energy structure. Biomass energy, as a kind of renewable energy with the characteristics of being easy to store and friendly to environment, has become the focus of China's energy development in the future. Affected by the advanced power generation technology and diversified geography environment, the biomass power generation projects show new features in recent years. Hence, it is necessary to evaluate the efficiency of biomass power generation industry by employing proper method with the consideration of new features. In this paper, the regional difference as a new feature of biomass power generation industry is taken into consideration, and the AR model is employed to modify the zero-weight issue when using data envelopment analysis (DEA) method to evaluate the efficiency of biomass power generation industry. 30 biomass power generation enterprises in China are selected as the sample, and the efficiency evaluation is performed. The result can provide some insights into the sustainable development of biomass power generation industry in China.

## 1. Introduction

As a developing country with large population, China is relatively short of energy storage. With the rapid development of economy and society, along with the industrialization and urbanization, the energy consumption in China will increase to 4 billion Mtce and the gap between energy production and consumption will reach the half of the total energy volume at the end of 2050. Meanwhile, China is still one of the few countries that relay on the fossil energy, which will cause severe environmental problems. Nowadays, the energy shortage and defective structure are pushing China to seek out and develop the substitute energy.

Being the one and only material and easy-stored renewable energy power, the biomass power is getting more and more attention globally. Till the end of 2012, the installation capacity of biomass power in USA has exceeded 10000 megawatt and the capacity has been planned to account for 50% of the total energy production at the end of 2050. Also, Germany aims to use the biomass power to meet 16% of the whole country's electricity demand, 10% of the heating demand, and 15% of EV power at the end of 2030.

According to the publicized IEA data in 2012, China has abundant biomass energy resource with the productivity of about 5 billion tons per year, and this amount ranks only next to the fossil energy resource. Hence, China can develop the biomass power generation industry. However, compared with the development of hydropower and nuclear, wind, and solar power, the biomass power in China was not fully taking off until the implement of* Renewable Energy Act* in 2006. Now, the installation capacity of biomass power in China has increased by 30% each year. According to the* Long-Term Renewable Energy Development Plan*, in 2020, the expected biomass power installation capacity will be 3000 kilowatts. With the industrial plan and policy incentive, the biomass power in China has entered the track of high-speed development.

Under this situation, if enough data can be collected and be used to evaluate the industry efficiency quantitatively, the evaluation result can provide basis for the industrial policy measurements to improve efficiency, which in return will accelerate the industrialization of biomass power in China and keep the development heading in the optimal direction. Therefore, to advance the sustainable development of China's biomass power generation industry, the efficiency of biomass power generation industry is evaluated by using data envelopment analysis (DEA) method. Meanwhile, taking the regional difference as a new feature of biomass power generation industry into consideration, the AR model is employed to modify the zero-weight issue when using DEA to evaluate the efficiency of biomass power generation industry.

The rest of this paper is organized as follows.  Section 2 reviews the existing literature related to biomass power generation industry.  Section 3 analyzes the characteristics of biomass power generation industry in China. Evaluation model and index selection are introduced in Section 4.  Section 5 gives the weight adjustment based on AR model. Efficiency evaluation on China's biomass power generation industry considering the regional effect is performed in Section 6.  Section 7 concludes this paper and gives some remarks.

## 2. Literature Review

Developed countries in Europe and USA started their research on biomass power in 1970s, and the early researches commonly focused on the energy conversion technology. With the subsequent development of biomass power industry, the studies and concerns shifted to the generating cost and industry efficiency. Hooper and Li come up with the countermeasures from the view of investment for the development of biomass power generation industry and found out the technology needed to be advanced and it was the best choice to industrialize and commercialize biomass power [1]. The biomass power took off late in China and the studies about the biomass generating technology were summed up and learned. Hence, the research on biomass power in China began with focusing on the economy and industry efficiency. Ma et al. started with the cost and came to the conclusion that only when the biomass was much cheaper than other fuels, the biomass power generation could gain better profits [2]. Huang overviewed the global developing tendency of biomass power and collected many projects to argue that the biomass was economical and would have great prospect [3]. Given this idea, Chen constructed the biomass-economy model consisting of technology, market, and public information to study biomass power generation of China by empirical analysis method [4].

After the implementation of* Renewable Energy Act*, the biomass power in China entered the high-speed industrial growth phase. Li and Shi pointed that, in this phase, the end-user should follow the “*consume more, then pay more*” rule and pay for the public benefits that were brought by the biomass power, so the industry could be supported [5]. This idea firstly and clearly included the environmental and social benefits into the outcome of biomass power generation. It created profitability for the biomass power generation in China and enlightened researchers to take in the comprehensive assessment methods to study the industry efficiency, not only to calculate the technical efficiency but also to evaluate the scale and overall efficiency. Christoph and Perrels used scenario analysis and input-output model to study how biomass power related to the CO_2_ emission [6]. Klevas et al. and St. Denis and Parker used DEA to analyze the technical efficiency of some common renewable technologies, including the biomass technology, respectively [7, 8]. P. Zhou and D. Q. Zhou meliorated the DEA model and made it suitable for overall efficiency assessment in China [9]. DEA method shows its advantage in evaluating the efficiency of biomass power generation industry, and Chinese scholars have applied it into China's empirical research. Zhao and Yan chose SWOT method to analyze the industry state of biomass power generation in China and thought that the advantages lied in the increasing electricity demand and changing electricity price, yet the industry was sensitive to local policy and local industrial environment [10]. Kautto and Peck also compared the regional and national biomass power plan, finding out that if the regional plan can cooperate with the national developing better, the whole biomass industry efficiency would take a step to a higher level [11]. In all, the regional or local situation indeed impacts the efficiency of biomass power generation industry.

As for China, with the multiple geographical features and unbalanced economy, the regional difference is distinct and regional effect is easy to influence the biomass industry evaluation; hence, the region needs to be considered as a key factor when an industry efficiency evaluation is performed. Other than that, when Cristobal and San Cristóbal used MCDEA to assess the efficiency of 13 projects located in different areas, some weights of the indexes equaled zero [12], which made the index lose the ability to contribute and led to inaccuracy of efficiency ranking. This paper considers the regional difference as a factor and uses AR model to modify the zero-weight problem and collects the data from the biomass power suppliers of different areas to evaluate the industry efficiency and then provides some insights into the sustainable development of China's biomass power generation industry.

## 3. Characteristics of Biomass Power Generation Industry in China

### 3.1. Conservative Rising Development Tendency

**Figure 1:**
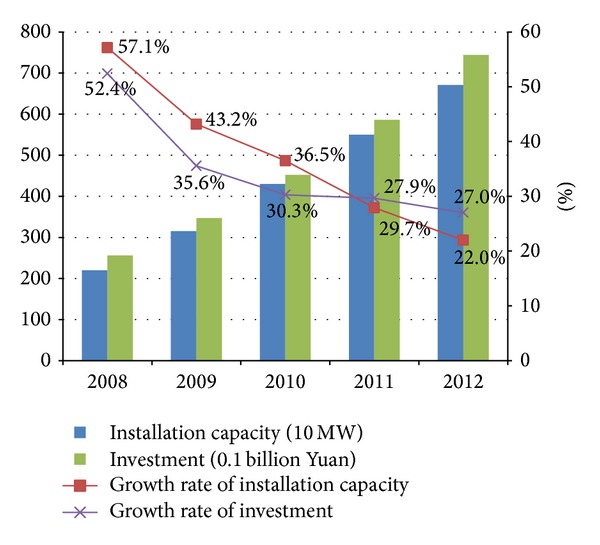
Industry scale and investment tendency of biomass power generation in China.

Since 2006, the biomass power generation industry has made huge progress. From 2008 to 2012, the installation capacity increased from 140 kilowatts to 550 kilowatts and the investment increased from 16.8 billion to 58.6 billion RMB, and both of these two indicators' average growth rate per year reached above 30%, which are shown in Figure 1.

The average growth rate per year of installation capacity and investment were both high during the past five years, but they were reducing each year, which show that the biomass power generation scale keeps growing but the growth rate is going down in China. On one hand, it fits the growth law; on the other hand, it shows that China is developing biomass power with positive but conservative attitude.

According to the* 12th Five-Year Energy Development Plan* and* Mid- and Long-Term Renewable Energy Development Plan*, the Chinese government plans to develop the installation capacity of biomass power to 1300 kilowatt and to invest over 90 billion RMB.

### 3.2. Single Impact from the Industry Chain

In China, the industry chain of biomass power generation is single. The upstream companies of biomass power generation are fuel suppliers and device manufactures, and the downstream one is the power-grid company. Because the fuel cost accounts for almost 60% of the whole biomass power generation cost in China, the main impact from the upstream is the fuel cost. According to the IEA's estimation, the potential biomass resource is abandon, which is listed in Table 1. Therefore, the fuel cost in China will not change dramatically in the near future.

As for the downstream company, because China now practices the “*full-amount-buy-in*” policy for renewable energy and the biomass power does not account for a large proportion, the electricity demand fluctuation will have little impact on the biomass power generation industry, and the main impact will mainly come from the feed-in price. According to the “*Agriculture and Forestry Biomass Price Policy*” issued by the* National Development and Reform Commission* of China (NDRC), for new biomass projects which have not determined its investor through biding, the feed-in price is set as 0.75 RMB per kWh (including the tax).

### 3.3. Distinct Regional Difference

It is distinct that the biomass power generation industry in China has regional distribution feature. Partly it is because different region has different types of biomass resource, and it is also due to the production characteristics of different biomass resource. For example, in South China, straw burning biomass power generation plants are built, because that area is rich of crop resources; however, in East China, the city area produces lots of municipal waste and garbage power plants are built.

**Figure 2:**
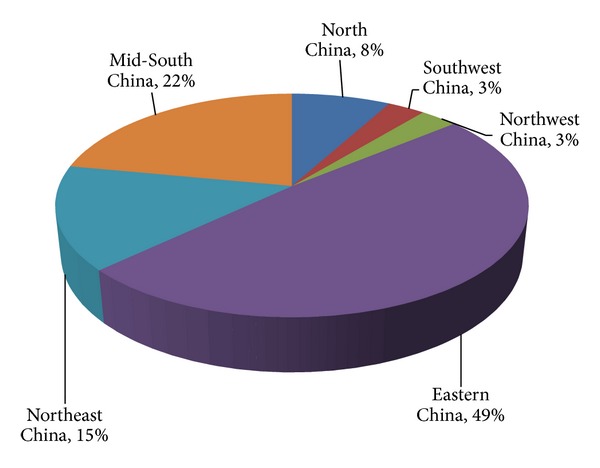
Biomass power distribution in China at the end of 2012.

Now, China's biomass power capacity is mainly distributed in Eastern China, and the following is Mid-South, Northeast, North, Southwest, and Northwest China, respectively. Till the end of 2012, the biomass power installation capacity distributed in the above areas is shown in Figure 2. This proportion will not change largely in the following years and be the same with the investment distribution.

## 4. Evaluation Model and Index Selection

DEA is a common model used for evaluating the industry efficiency and usually can produce feasible and optimal solution, yet due to the features of constrained linear programming, the optimal solution is not exclusive. Besides, the weight of each index calculated through linear constraint is the preference value that maximizes the objective function. When a DMU has one or some outstanding indexes, the influence caused by the other indexes will be covered, which will lead to the false optimization and cause discrepancy with the practical situation.

To settle the above issue, when DEA method is used to evaluate the efficiency of biomass power generation industry, the AR model is employed to modify the zero-weight issue. The specific step of evaluation on the efficiency of biomass power generation industry in China is as follows. Firstly, the BC^2^ model is used to evaluate the sample data and make the basic DEA estimation; then, according to the calculated result, it imposes restrictions on the weight value interval to adjust the outstanding index; finally, the AR model is used to evaluate the regional efficiency of biomass power generation industry in China, which can make the evaluation more practical and usable.

According to the current situation and the characteristics of biomass power industry in China, with the consideration of production factor price's complexity, volatility, and externality, the installation capacity, investment, and annual fuel consumption are selected as the input indexes and the annual generating capacity, annual emission reduction, and annual coal conservation as the output indexes.

**Figure 3:**
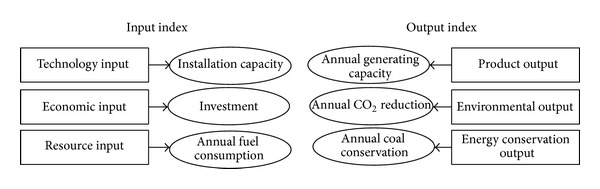
Input and output indexes.

As shown in Figure 3, the installation capacity relates to the technology input; the investment relates to the input poured in equipment and infrastructure construction. Because the variable cost of biomass power generation in China is basically affected by the fuel price, the annual fuel consumption is also selected to reflect the resource input. Obviously, the annual generating capacity, emission reduction, and coal conservation can be considered as the reflection of product, environmental benefit, and energy conservation benefit, respectively.

According to the reported statistics, there are about 100 biomass power generation enterprises in China now. This paper samples the related data from 30 enterprises located in different regions of China and uses it to make regional industry efficiency evaluation. Because the data is from commercial survey, the names of these sampled enterprises are replaced by code name as E1, E2, E3, and so on to protect the business information.

## 5. Weight Adjustment Based on AR Model

Firstly, the basic DEA evaluation using the data from 30 Chinese enterprises based on BC^2^ model is performed. Set *v*_1_, *v*_2_, *v*_3_ as the weights of installation capacity, investment, and annual fuel consumption, respectively, and *u*_1_, *u*_2_, *u*_3_ as the weights of annual generating capacity, emission reductions, and coal conservation, respectively. The calculation result is listed in Table 2.

As listed in Table 2, most of the DMU had zero values related to *u*_1_ and *u*_3_, which indicates that most of the enterprises' annual generating capacity and coal conversation indexes cannot play a part in the efficiency evaluation. That would cause the evaluation inaccuracy.

According to the survey, 78% of the enterprises consider that among the input indexes, the significance order is the installation capacity, investment, and annual fuel consumption. Hence, based on the calculation result, the input index weights are restricted as

(1) $$\begin{array}{c} 0.8\leq \frac{v_{2}}{v_{1}}\leq 1,\;\;0.2\leq \frac{v_{3}}{v_{1}}\leq 0.5. \end{array}$$

For the output indexes, 84% of the enterprises consider that the generating capacity is the most significant index. Because all the regions in China are under the great pressure of energy conservation and emission reduction, the enterprises believe that the annual emission reduction is equally important with the annual coal conservation. So, the output index weights are restricted as

(2) $$\begin{array}{c} 1.1\leq \frac{u_{2}}{u_{1}}\leq 1.3,\;\;1\leq \frac{u_{3}}{u_{1}}\leq 1. \end{array}$$

Using the restricted weights above to perform the AR evaluation, the calculation result is listed in Table 3. Compared with the DMU weight listed in Table 2, all the weights of DMU do not equal zero. As listed in Table 3, the DEA of E7, E10, E16, E20, E24, E26, and E29 changes from efficiency to nonefficiency, respectively, but the DEA of E6, E28, and E30 remains efficient. This indicates E6, E28, and E30 have higher efficiency by any evaluation method, and they are more suitable for being as the reference set. The weight modification of AR model has good performance, which can be employed to evaluate the industrial efficiency of different regions.

## 6. Efficiency Evaluation under Regional Effect 

The sampled 30 biomass power generation enterprises are located in Eastern, North, Southwest, Mid-South, Northwest, and Northeast China. Because the biomass resource endowment and generating technology are not the same in these regions, a general evaluation will be affected by the regional effect and lose accuracy to some extent. Hence, eliminating the natural resource and environmental factors caused by region difference can significantly improve the accuracy of industry efficiency evaluation and reveal the real development status in China.

From the view of external industrial environment, East, South, and Southwest China have the similar power grid condition and the local consumption habits are more conducive to the operation of biomass power generation enterprises, while the Northeast, North, and Northwest China do not have preferable natural biomass resource. Hence, this paper divides China into North and South parts. The power generation enterprises located in Northern China were evaluated within the Northern area, and the ones located in Southern China were evaluated within the nationwide area.

Based on the two divided parts and restricted weights above, the AR model is used again to make separate calculation, and the result is listed in Table 4. Compared to the calculation results, DEA-efficiency enterprises under AR model include E6, E28, and E30 before the region is divided. As a reference set, there is not much information to refer to. After the region is divided, DEA-efficiency units become {E28, E30, E4, E6, E7, and  E26}.

After the region was divided, E4, E7, and E26 located in North part actually have higher efficiency, but being bound by the external environment of biomass power generation industry, the evaluated indicators' values are affected, and then the actual efficiency cannot be reflected under the conditions of both limited weight and nondivided regions. From a management perspective, there is no redundancy of the above three enterprises in terms of resources allocation on input and output, and the gap between projection value and actual value is narrow, which indicates that the DEA may be efficient, and the production efficiency can be reflected after eliminating the regional effect and modifying the reference set. However, under the same modification condition, the evaluated efficiency values of E17, E18, and E29 are still below 0.5, which indicate that there is room for efficiency improvement for these three enterprises.

Without considering the regional effect, the evaluation result on all the enterprises by employing AR model shows that the efficiency mean value of enterprises located in South part is 0.59, and the one of enterprises located in North part is 0.49, which indicates that the industrial efficiency of enterprises located in South part is much larger than that in North part. However, taking the regional effect into consideration, the industrial efficiency of enterprises located in North part increases to 0.77, which is larger than that in South part. Therefore, two conclusions can be safely drawn: (1) both biomass resource and power grid environment can cause a certain impact on the efficiency evaluation of biomass power generation industry and (2) from the management perspective, the management efficiency of biomass power generation enterprises located in North part is higher than that in South part, but the cost of biomass fuel is much higher, and the management efficiency can be benefited from the environmental improvement of market and industry.

## 7. Conclusions and Remarks

Biomass energy resource is rich in China, which has great development potentiality. However, the distribution of biomass energy resource is uneven, and different regions have different resource reserves. Henan and Shandong provinces are the main distribution regions of biomass energy, and the regional industrial efficiencies are higher than other regions due to the good environment of fuel resource market and power grid. Meanwhile, the potential distribution of biomass energy resource is complementary with that of conventional primary energy source to some extent, which makes the regions with low reserves of primary energy source have great potential for utilizing biomass energy. The DEA and AR models are employed to evaluate the efficiency of China's biomass power generation industry in this paper, and some evaluation results are obtained. For North part (except northeast region), the overall efficiency is lower than that of South part due to the high biomass fuel cost. However, purely from a management perspective, the management level of North part is higher than that in South part. Therefore, the biomass energy industry in North part can be further developed with the improvement of market and industry environment.

To be specific, the biomass generation industry in Eastern China develops well and its industrialization level is high. A great many biomass power generation enterprises have flocked together in Eastern China. However, in the long run, the future development priority should be changed from increasing quantity to advancing quality due to the limited resource volume. The biomass energy resource in South-central China is abundant and has great development potential, which is the main region of biomass power generation industry in the future. The development of biomass energy industry in Northeast China is stable, and the straw power generation is the main development direction in the future. The straw direct-fired power generation and garbage incineration power generation are the main power generation types in North China, and the power generation structure will not change largely in the future. The development of biomass power generation industry in Northwest China is relatively backward, but the power generation capacity has a good prospect because the biomass energy resource in this region is relatively rich. The garbage incineration power generation is the main power generation type in Southwest China due to the regional energy development plan, and the straw power generation has great development space due to the rich crop straw and large electricity shortage.

## Figures and Tables

**Table 1:** The potential biomass resource in China estimated by IEA (unit: 10^8^ Mtce).

Biomass resource type	2010	2020	2030	2050
Present biomass resource	2.8	2.8	2.8	2.8
Newly added organic waste	0.6	1.7	2.2	2.7
Present woodland growth	0.05	0.3	0.7	1.37
New ground marginal product	0.05	0.3	1	2
Total potential	**3.5**	**5.1**	**6.7**	**8.9**

**Table 2:** Evaluation result of BC^2^ model.

Enterprises	*v*_1_	*v*_2_	*v*_3_	*u*_1_	*u*_2_	*u*_3_	*θ*
E1	0.0148	0.3188	0.0000	0.0000	0.0394	0.0000	0.8800
E2	0.0069	0.2774	0.0030	0.0000	0.0515	0.0000	0.8640
E3	0.0082	0.0352	0.0408	0.0000	0.0791	0.0000	0.8860
E4	0.0004	0.0213	0.0304	0.0000	0.0760	0.0000	0.9300
E5	0.0100	0.2272	0.0006	0.0000	0.0216	0.0799	0.7130
E6	0.0146	0.3958	0.0437	0.0000	0.0066	0.0265	1.0000
E7	0.0182	0.5460	0.0021	0.0000	0.0144	1.5216	1.0000
E8	0.0000	0.0524	0.0406	0.0000	0.0562	0.0000	0.6590
E9	0.0000	0.0614	0.0476	0.0000	0.0659	0.0000	0.9230
E10	0.0125	0.2699	0.0001	0.0000	0.0272	0.0375	1.0000
E11	0.0023	0.3050	0.0052	0.0000	0.0782	0.0000	0.7700
E12	0.0000	0.0704	0.0650	0.0000	0.0743	0.0000	0.8650
E13	0.0070	0.0301	0.0350	0.0000	0.0678	0.0000	0.8660
E14	0.0101	0.2324	0.0000	0.0000	0.0218	0.0747	0.7280
E15	0.0070	0.0301	0.0350	0.0000	0.0678	0.0000	0.7950
E16	0.0087	0.4416	0.0014	0.0000	0.0530	0.8831	1.0000
E17	0.0163	0.0000	0.0296	0.0000	0.0000	0.6176	0.8160
E18	0.0006	0.0315	0.0450	0.0000	0.1125	0.0000	0.8140
E19	0.0062	0.0267	0.0310	0.0000	0.0601	0.0000	0.8340
E20	0.0177	0.0753	0.0114	0.0008	0.3122	5.0080	1.0000
E21	0.0112	0.2553	0.0007	0.0000	0.0242	0.0897	0.8130
E22	0.0126	0.2905	0.0000	0.0000	0.0272	0.0933	0.8050
E23	0.0070	0.0301	0.0349	0.0000	0.0677	0.0000	0.6070
E24	0.0024	0.0109	0.0612	0.0000	0.0897	0.0749	1.0000
E25	0.0067	0.0715	0.0228	0.0000	0.0497	0.0000	0.7000
E26	0.0121	0.0793	0.0049	0.0005	3.5683	28.757	1.0000
E27	0.0071	0.2846	0.0031	0.0000	0.0528	0.0000	0.7330
E28	0.0991	0.0164	0.0064	0.0002	11.001	14.466	1.0000
E29	0.0066	0.0863	0.0035	0.0002	1.3310	98.604	1.0000
E30	0.1818	0.0051	0.0038	0.0000	0.0043	0.0067	1.0000

**Table 3:** Weight constraint of AR model and evaluation result.

Enterprises	*v*_1_	*v*_2_	*v*_3_	*u*_1_	*u*_2_	*u*_3_	*u*_0_	*θ*	*θ*(BC^2^)	*v*_2_/*v*_1_	*v*_3_/*v*_1_	*u*_2_/*u*_1_	*u*_3_/*u*_1_
E1	0.0334	0.0334	0.0167	0.0253	0.0329	0.0253	0.2637	0.6021	0.8800	1	0.5	1.300395	1
E2	0.0226	0.0226	0.0113	0.0199	0.0219	0.0199	0.1734	0.4856	0.8640	1	0.5	1.100503	1
E3	0.0244	0.0244	0.0122	0.0215	0.0237	0.0215	0.1874	0.4768	0.8860	1	0.5	1.102326	1
E4	0.0206	0.0206	0.0103	0.0181	0.0199	0.0181	0.1577	0.5284	0.9300	1	0.5	1.099448	1
E5	0.0208	0.0208	0.0104	0.0183	0.0202	0.0183	0.1597	0.4249	0.7130	1	0.5	1.103825	1
E6	0.0583	0.0564	0.0291	0.0024	0.0029	0.0024	0.9845	1	1.0000	0.96741	0.499142	1.208333	1
E7	0.0493	0.0493	0.0246	0.007	0.0077	0.007	0.8048	0.8714	1.0000	1	0.498986	1.1	1
E8	0.0202	0.0202	0.0101	0.0025	0.0033	0.0025	0.3295	0.3534	0.6590	1	0.5	1.32	1
E9	0.0241	0.0241	0.012	0.0212	0.0233	0.0212	0.1844	0.464	0.9230	1	0.497925	1.099057	1
E10	0.0263	0.0263	0.0131	0.0231	0.0254	0.0231	0.2015	0.5156	1.0000	1	0.498099	1.099567	1
E11	0.0228	0.0228	0.0114	0.0201	0.0221	0.0201	0.175	0.4705	0.7700	1	0.5	1.099502	1
E12	0.0294	0.0294	0.0147	0.0037	0.0047	0.0037	0.4808	0.5247	0.8650	1	0.5	1.27027	1
E13	0.0233	0.0233	0.0116	0.0205	0.0225	0.0205	0.1783	0.4795	0.8660	1	0.497854	1.097561	1
E14	0.0208	0.0208	0.0104	0.0183	0.0202	0.0183	0.1597	0.385	0.7280	1	0.5	1.103825	1
E15	0.0233	0.0233	0.0116	0.0205	0.0225	0.0205	0.1783	0.4457	0.7950	1	0.497854	1.097561	1
E16	0.0324	0.0324	0.0162	0.0245	0.0319	0.0245	0.2555	0.6464	1.0000	1	0.5	1.302041	1
E17	0.0264	0.0264	0.0132	0.0232	0.0255	0.0232	0.202	0.487	0.8160	1	0.5	1.099138	1
E18	0.0235	0.0235	0.0117	0.0178	0.0231	0.0178	0.185	0.4723	0.8140	1	0.497872	1.297753	1
E19	0.0198	0.0198	0.0099	0.0174	0.0192	0.0174	0.1518	0.4202	0.8340	1	0.5	1.103448	1
E20	0.0231	0.0231	0.0116	0.0204	0.0224	0.0204	0.1775	0.47	1.0000	1	0.502165	1.098039	1
E21	0.0261	0.0261	0.0131	0.023	0.0253	0.023	0.2002	0.4962	0.8130	1	0.501916	1.1	1
E22	0.0257	0.0257	0.0129	0.0226	0.0249	0.0226	0.1971	0.465	0.8050	1	0.501946	1.10177	1
E23	0.0232	0.0232	0.0116	0.0029	0.0037	0.0029	0.3796	0.4175	0.6070	1	0.5	1.275862	1
E24	0.0207	0.0207	0.0104	0.0183	0.0201	0.0183	0.1591	0.4289	1.0000	1	0.502415	1.098361	1
E25	0.0219	0.0219	0.0109	0.0193	0.0212	0.0193	0.1678	0.445	0.7000	1	0.497717	1.098446	1
E26	0.0147	0.0147	0.0073	0.0129	0.0142	0.0129	0.1126	0.4155	1.0000	1	0.496599	1.100775	1
E27	0.0225	0.0225	0.0112	0.017	0.0221	0.017	0.1773	0.4279	0.7330	1	0.497778	1.3	1
E28	0.0501	0.0488	0.0112	11.1859	13.0286	11.1859	−311.42	1	1.0000	0.974052	0.223553	1.164734	1
E29	0.012	0.012	0.006	0.0105	0.0116	0.0105	0.0918	0.3321	1.0000	1	0.5	1.104762	1
E30	0.0781	0.0684	0.0202	0.0305	0.0365	0.0305	0.6179	1	1.0000	0.8758	0.258643	1.196721	1

**Table 4:** Evaluation result based on AR model after the region was divided.

Enterprises	*v*_1_	*v*_2_	*v*_3_	*u*_1_	*u*_2_	*u*_3_	*u*_0_	*θ*	*θ*(AR)	Part
E1	0.0334	0.0334	0.0167	0.0253	0.0329	0.0253	0.2637	0.6021	0.6021	South
E11	0.0228	0.0228	0.0114	0.0201	0.0221	0.0201	0.175	0.4705	0.4705	South
E12	0.0294	0.0294	0.0147	0.0037	0.0047	0.0037	0.4808	0.5247	0.5247	South
E16	0.0324	0.0324	0.0162	0.0245	0.0319	0.0245	0.2555	0.6464	0.6464	South
E17	0.0264	0.0264	0.0132	0.0232	0.0255	0.0232	0.202	0.487	0.487	South
E18	0.0235	0.0235	0.0117	0.0178	0.0231	0.0178	0.185	0.4723	0.4723	South
E7	0.0225	0.0225	0.0112	0.017	0.0221	0.017	0.1773	0.4279	0.4279	South
E28	0.0501	0.0488	0.0112	11.1859	13.0286	11.1859	−311.42	1	1	South
E29	0.012	0.012	0.006	0.0105	0.0116	0.0105	0.0918	0.3321	0.3321	South
E30	0.0781	0.0684	0.0202	0.0305	0.0365	0.0305	0.6179	1	1	South
E2	0.0226	0.0226	0.0113	0.0586	0.0645	0.0586	−0.101	0.8187	0.4856	North
E3	0.0248	0.0198	0.0124	0.0632	0.0696	0.0632	−0.1071	0.7434	0.4768	North
E4	0.0247	0.0204	0.0061	0.156	0.1832	0.156	−2.3672	1	0.5284	North
E5	0.0256	0.0256	0.0051	0.051	0.0561	0.051	−0.0279	0.7095	0.4249	North
E6	0.0667	0.059	0.0161	0.0172	0.0205	0.0172	0.8887	1	1	North
E7	0.0519	0.0456	0.0203	0.0493	0.0594	0.0493	0.4959	1	0.8714	North
E8	0.0205	0.0164	0.0102	0.0522	0.0574	0.0522	−0.0884	0.5377	0.3534	North
E9	0.0244	0.0195	0.0122	0.0622	0.0685	0.0622	−0.1054	0.7166	0.464	North
E10	0.0315	0.0315	0.0063	0.0626	0.0689	0.0626	−0.0343	0.8161	0.5156	North
E13	0.0233	0.0233	0.0116	0.0603	0.0663	0.0603	−0.1038	0.7834	0.4795	North
E14	0.0256	0.0256	0.0051	0.051	0.0561	0.051	−0.0279	0.5985	0.385	North
E15	0.0236	0.0189	0.0118	0.0601	0.0661	0.0601	−0.1019	0.6841	0.4457	North
E19	0.0198	0.0198	0.0099	0.0513	0.0565	0.0513	−0.0884	0.7021	0.4202	North
E20	0.0235	0.0188	0.0117	0.0599	0.0659	0.0599	−0.1015	0.759	0.47	North
E21	0.0324	0.0259	0.0065	0.0631	0.0694	0.0631	−0.0306	0.7823	0.4962	North
E22	0.0318	0.0318	0.0064	0.0633	0.0696	0.0633	−0.0346	0.7152	0.465	North
E23	0.0236	0.0189	0.0118	0.0519	0.0675	0.0519	−0.0901	0.5905	0.4175	North
E24	0.0212	0.017	0.0106	0.0541	0.0595	0.0541	−0.0916	0.7077	0.4289	North
E25	0.0262	0.0262	0.0052	0.052	0.0572	0.052	−0.0285	0.7207	0.445	North
E26	0.0154	0.0146	0.0061	12.8458	15.3004	12.8458	−318.719	1	0.4155	North
